# PEMOCS: Evaluating the effects of a concept-guided, PErsonalised, MOtor-Cognitive exergame training on cognitive functions and gait in chronic Stroke—study protocol for a randomised controlled trial

**DOI:** 10.1186/s13063-024-08283-7

**Published:** 2024-07-04

**Authors:** S.K. Huber, R.H. Knols, J.P.O. Held, M. Betschart, E.D. de Bruin

**Affiliations:** 1 Physiotherapy Occupational Therapy Research Center, Directorate of Research and Education, University Hospital Zurich, Zurich, Switzerland; 2 Motor Control and Learning Group, Institute of Human Movement Sciences and Sport, Department of Health Sciences and Technology, ETH Zurich, Zurich, Switzerland; 3 Rehabilitation Center Triemli Zurich, Valens Clinics, Zurich, Switzerland; 4 Department of Health, OST – Eastern Swiss University of Applied Sciences, St. Gallen, Switzerland; 5 Institute of Therapy and Rehabilitation, Kantonsspital Winterthur, Winterthur, Switzerland; 6 Department of Neurobiology, Care Sciences and Society, Division of Physiotherapy, Karolinska Institutet, Stockholm, Sweden

**Keywords:** Stroke, Training, Gaming, Virtual reality, Rehabilitation, Gait, Mobility, Cognition, Protocol

## Abstract

**Background:**

Many stroke survivors remain with residual cognitive and motor impairments despite receiving timely acute and sub-acute rehabilitation. This indicates that rehabilitation following stroke should be continuous to meet the needs of individual stroke patients. Both cognitive and motor functions are essential for mastering daily life and, therefore, should be aimed at with rehabilitation. Exergames, motor-cognitive exercises performed using video games, are an auspicious method to train both motor and cognitive functions and at the same time may foster the long-term motivation for training. This study aims to assess the effect of concept-guided, personalised, motor-cognitive exergame training on cognitive and motor functions in chronic stroke survivors.

**Methods:**

This study is a single-blinded, randomised controlled trial. Assessments are performed at baseline, after a 12-week intervention, and at a 24-weeks follow-up. Chronic stroke patients (≥ 18 years old, ≥ 6 months post-stroke) able to stand for 3 min, independently walk 10 m, follow a two-stage command, and without other neurological diseases apart from cognitive deficits or dementia are included. Participants in the intervention group perform the exergame training twice per week for 30 (beginning) up to 40 (end) minutes additionally to their usual care programme. Participants in the control group receive usual care without additional intervention(s). Global cognitive functioning (total Montreal Cognitive Assessment (MoCA) score) is the primary outcome. Secondary outcomes include health-related quality of life, specific cognitive functions, single- and dual-task mobility, and spatiotemporal gait parameters. The target sample size for this trial is 38 participants. Linear mixed models with the post-outcome scores as dependent variables and group and time as fixed effects will be performed for analysis.

**Discussion:**

Superior improvements in global cognitive functioning and in the abovementioned secondary outcomes in the intervention group compared to the control group are hypothesised. The results of this study may guide future design of long-term rehabilitation interventions after stroke.

**Trial registration:**

ClinicalTrials.gov (NCT05524727). Registered on September 1, 2022.

**Supplementary Information:**

The online version contains supplementary material available at 10.1186/s13063-024-08283-7.

## Administrative Information

Note: the numbers in curly brackets in this protocol refer to SPIRIT checklist item numbers. The order of the items has been modified to group similar items (see http://www.equator-network.org/reporting-guidelines/spirit-2013-statement-defining-standard-protocol-items-for-clinical-trials/).

**Table:** 

Title {1}	PEMOCS: Evaluating the effects of a concept-guided, PErsonalized, MOtor-Cognitive exergame training in chronic Stroke – study protocol for a randomized controlled trial
Trial registration {2a and 2b}	clinicaltrials.gov (NCT05524727)
Protocol version {3}	#5, 20/10/2023
Funding {4}	This study is supported by grants of the USZ Innovation Pool (https://usz-foundation.com/en/usz-innovation-pool/) and the Swiss Association of Physiotherapy (physioswiss).
Author details {5a}	Huber S.K., Knols R.H., Held J.P.O., Betschart M., de Bruin, E.D.
Name and contact information for the trial sponsor {5b}	Rudolf H. Knols, PhD, University Hospital Zurich, Rämistrasse 100, CH-8091 Zurich, ruud.knols@usz.ch
Role of sponsor {5c}	Oversight of Good Clinical Practice regulatory requirements

## Introduction

### Background and rationale {6a}

Stroke is a dominant global health burden and a major cause of long-term disability in adults [1–4]. In Switzerland, approximately 20,000 persons suffer a stroke each year [5]. A stroke can cause motor and cognitive impairments [6, 7]. The most common motoric consequence of stroke is hemiparesis, a unilateral paralysis or weakness of either one or both extremities [8]. It occurs in up to 80% of patients with stroke [9]. Hemiparesis typically leads to gait and balance impairments [10, 11], which can restrict mobility and independent ambulation [12]. Common cognitive impairments after stroke include deficits in executive functions, attention, spatial perception, and psychomotor processing speed [13, 14]. These cognitive impairments occur in a comparable frequency as motor impairments; depending on the specific cognitive function, 30 to 90% of stroke survivors suffer from cognitive impairment [7, 15, 16].

Residual impairments, consequences from the stroke lasting after the acute and sub-acute rehabilitation phase, are common after stroke [17, 18]. This is especially true for cognitive impairments. It is striking in this context that cognitive impairments have so far gained much less attention in research compared with motor impairments notwithstanding the fact patients mentioning ‘What are the best ways to improve cognition after stroke?’ being one of their foremost research priorities in relation to life after stroke [19]. Remaining cognitive deficits are often responsible for limited independence and quality of life in patients, who have regained good motor functioning and activities-of-daily-living ability [20]. The optimal treatment of cognitive deficits in chronic stroke patients is a clearly identifiable research gap [17, 21].

Motor-cognitive training is a comprehensive rehabilitation method that combines motor and cognitive training [22]. Combined motor-cognitive trainings may be an auspicious method to tackle this research gap. Motor-cognitive trainings can either be performed sequentially (first motor training component, then cognitive training component, or vice versa) or simultaneously (both task components executed at the same time) [22, 23]. Simultaneous motor-cognitive trainings, where both tasks are executed concurrently, may have the highest relevance for daily life, because daily life almost exclusively sets combined challenges [22]. Motor and cognitive functions have been shown to share structural and functional roots [24]. Fittingly, the ‘guided plasticity facilitation’ model suggests that motor-cognitive training can lead to additional benefits due to interaction effects of the two components [25, 26]. Confirming the theory, multiple systematic reviews in healthy older adults found that combined motor-cognitive trainings were superior in improving motor, cognitive, and dual-task functions [27–35]. In (chronic) stroke, however, there is less and yet unclear evidence. While first systematic reviews report beneficial effects of motor-cognitive over single interventions on balance and gait [36, 37], the effects on cognitive functions remain unclear to date [37–39].

The most promising type of simultaneous motor-cognitive trainings may be exergames [37], cognitively demanding video games, which require the player to be physically active to complete the gaming tasks [40, 41]. Gamification of training and the use of virtual reality can increase the motivation for and adherence to exergame training [42–44], while also giving the training ecological validity [45]. In healthy older adults, exergames have been shown to improve motor and dual-task functions [46–51], while potential has been reported for improving cognitive functions [52–56]. In other neurological populations, exergames showed beneficial effects on balance, mobility, and walking capacity [57–62], while the evidence for improving cognitive functions with exergames is yet scarce and inconsistent [63–66]. In (chronic) stroke, exergames have been found a suitable adjunct to conventional rehabilitation for improving motor functions [67–69]. However, little is yet known about the effect of motor-cognitive exergames on cognitive functions in chronic stroke, so further studies are needed [37]. Moreover, in most previous studies including stroke survivors, motor-cognitive exergame interventions were applied without systematically considering training principles, especially personalised progression [37]. It is known, however, that applying training principles and personally tailoring the training to the individual is important for the success of any training intervention [70, 71]. Therefore, we developed a training concept considering the FITT-VP (frequency, intensity, time, type, volume, progression) training principles in combination with neuroplasticity and motor learning principles [70, 72]. Applying this concept, it is the aim of this study to investigate if adding motor-cognitive exergame training to usual care has a beneficial effect on the long-term rehabilitation of chronic stroke survivors and if global cognitive functioning and secondary outcomes can be improved compared to a control group who does not receive the exergame training.

### Objectives {7}

To address the gaps of knowledge regarding rehabilitation of cognitive functions and effects of motor-cognitive exergames in chronic stroke, the primary objective of this study is to evaluate the effect of a 12-week concept-guided, personalised, motor-cognitive exergame training when added to usual care, in comparison to the effect of usual care alone on global cognitive functioning in chronic stroke survivors.

The secondary objectives of this study are to explore (1) the acute and (2) the persistent effect up to a 12-week follow-up of the exergame intervention on health-related quality of life, specific cognitive functions (sub-functions of attentional, executive, and visuospatial functions), single- and dual-task mobility, and spatiotemporal parameters in chronic stroke patients.

### Trial design {8}

This study is a randomised, controlled trial (RCT) with two parallel arms, investigating the superiority of a concept-guided, personalised, motor-cognitive exergame training added to usual care over usual care alone. The trial employs a single-blinded approach, with outcome assessors being unaware of group assignments. The randomisation is performed with a 1:1 ratio using stratification by sex (female or male [73]) and cognitive impairment (MoCA ≥ 24 or MoCA < 24 [74]).

## Methods: participants, interventions, and outcomes

### Study setting {9}

This study takes place in different academic and rehabilitation hospitals in the Canton of Zurich, Switzerland.

### Eligibility criteria {10}

#### Inclusion criteria

Participants fulfilling all the following inclusion criteria are eligible for the study:

- Adults (≥ 18 years) with chronic stroke (≥ 6 months post-stroke, ischemic or haemorrhagic [75])
- Able to stand for 3 min and walk 10 m, Functional Ambulation Category (FAC) ≥ 3
- Able to follow a two-stage command (no MoCA threshold for inclusion was set to prevent exclusion of persons with subtle or specific cognitive deficits [76])
- Able to give informed consent as documented by signature

#### Exclusion criteria

Participants fulfilling one or more of the following exclusion criteria are not eligible for the study:

- Unable or not willing to give informed consent
- Having been diagnosed with other neurological diseases (e.g. Parkinson’s Disease, multiple sclerosis), except cognitive deficits or dementia
- Clinical contra-indications for the study intervention
- Unable to follow the study intervention or the test for the primary endpoint (MoCA), e.g. due to a neglect, aphasia, or other language problems
- Overlapping enrolment in another clinical trial

### Who will take informed consent? {26a}

Movement scientists and therapists who are part of the study team and have been trained for this task obtain written informed consent from participants, after they have been written and orally informed about the study, its benefits and risks, and their rights and had at least 24 h for consideration.

### Additional consent provision for collection and use of participant data and biological specimens {26b}

The consent includes collection and use of individually identifiable participant data for the study procedures (through members of the study team only) and for audit trials by the cantonal ethical committee and other local authorities. Moreover, participants consent that their de-identified data is used for the analysis and publication of the study results. This study does not involve collecting biological specimens for storage.

## Interventions

### Explanation for choice of comparators {6b}

Participants in the control group continue with their usual care and receive no additional intervention. They are called once a week to align for contact to the study team and to gather their physical and cognitive activity data (see the ‘ Intervention and further activity outcomes’ section). This comparator was chosen as the aim of this study is to determine the effect of additional motor-cognitive exergame training. This aim was based on systematic reviews, which recommend the application of exergame training in addition to usual care, aiming at increasing the amount of rehabilitation offered to patients [63, 67, 77].

### Intervention description {11a}

**Fig. 1:**
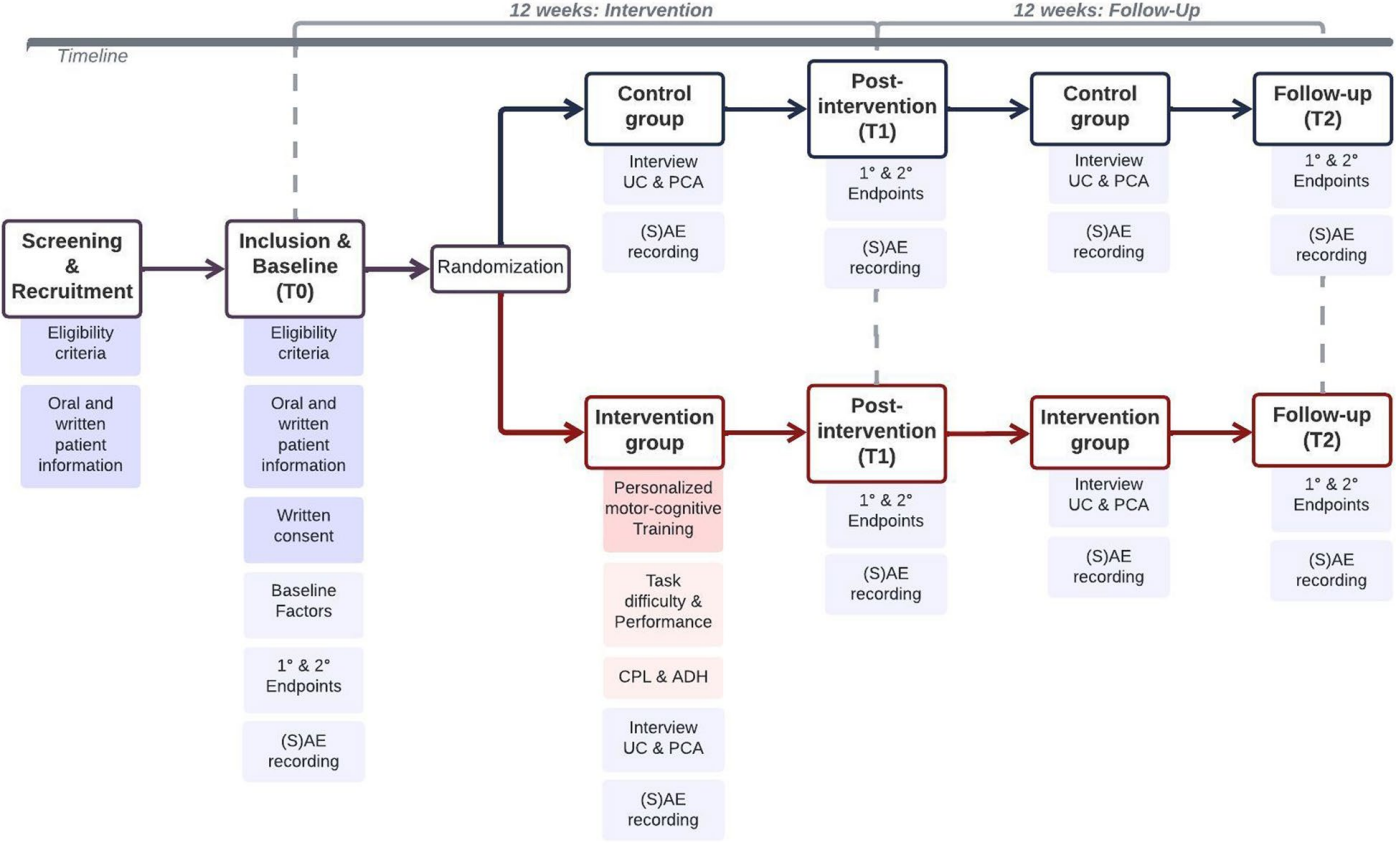
Study flow. Overview of the study procedures. ADH, adherence; CPL, compliance; PCA, physical and cognitive activities; (S)AE, (serious) adverse event; UC, usual care

The intervention group receives concept-guided, personalised, motor-cognitive exergame training additionally to usual care. The personalised motor-cognitive exergame training for stroke (PEMOCS) concept, which guides the intervention, was developed specifically for this study and, in accordance with the Modified Consensus on Exercise Reporting Template (CERT) for Therapeutic Exercise Interventions [78, 79], published elsewhere [80]. It determines the training dosage with regards to frequency, intensity, time, type, and volume of the exercises, which are based on recommendations from scientific literature for motor-cognitive and exergame trainings in chronic stroke and healthy older adults. Participants train twice a week for 12 weeks (intervention period, see Fig. 1). Training sessions last between 30 (beginning) and 40 (end) minutes, progressing in duration for 2 min every second week and resulting in 840 min total planned training time (see Table 1). Additionally, the PEMOCS concept is designed to provide personalised progression and variability in training considering principles for neuroplasticity, motor learning, and training [80–83]. It was developed based on Gentile’s Taxonomy for motor learning [84] and tested for its feasibility in the target population [85]. In short, motor and cognitive tasks of the exergame training are allocated to various difficulty levels along a skill-progression scheme with three dimensions. Based on the participants’ subjective ratings of their perceived motor-cognitive task difficulty and their perceived performance (see ‘Intervention outcomes’), progression through the difficulty levels is determined individually for each participant. Therefore, the training is personalised within a standardised progression scheme. Additionally, variability rules ensure variation in training tasks.

**Table 1:** Training variables of the intervention, describing the frequency, time, volume per week, and total volume of the concept-guided, personalised, motor-cognitive exergame training

**Time point**	**Frequency** [per week]		**Time** [min/session]		**Volume/week** [min]	**Total volume** [min]
Weeks 1–2	2	x	30	=	60	
Weeks 3–4	2	x	32	=	64	
Weeks 5–6	2	x	34	=	68	
Weeks 7–8	2	x	36	=	72	
Weeks 9–10	2	x	38	=	76	
Weeks 11–12	2	x	40	=	80	**840**

**Fig. 2:**
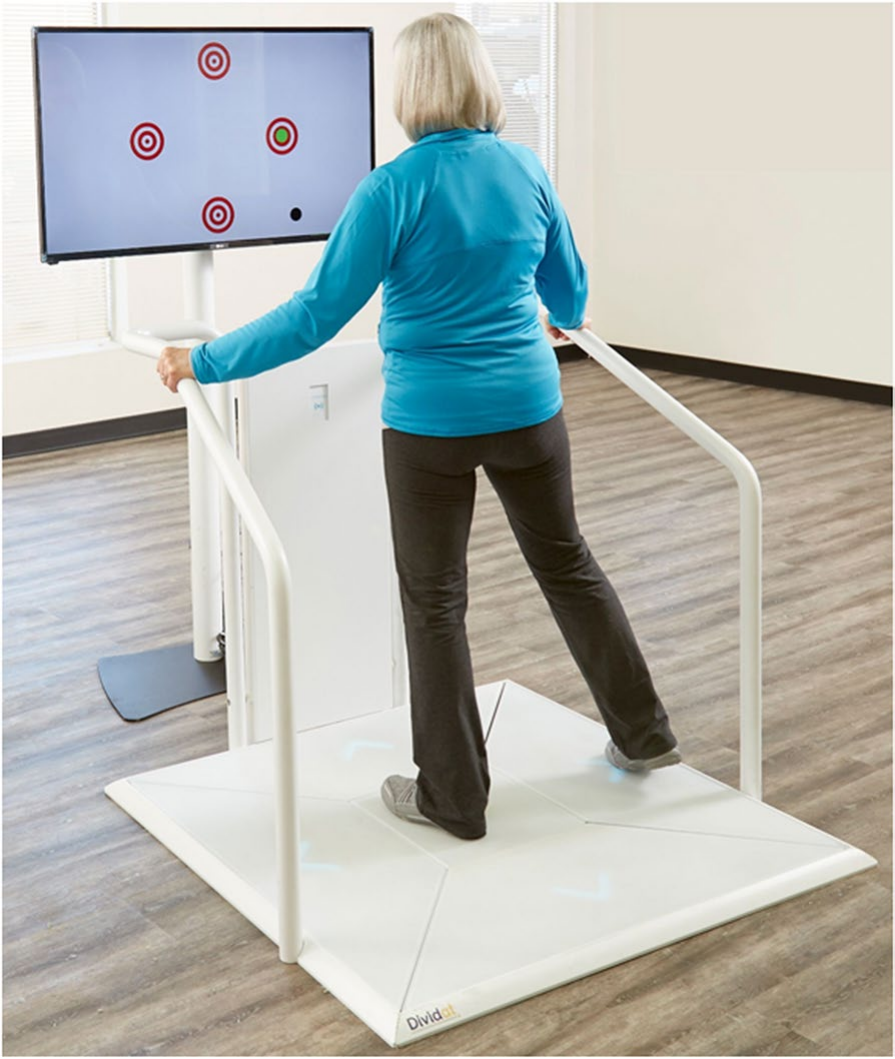
Study device: Dividat Senso in action. Pressure-sensitive plate with handrails on three sides and screen on head-height of the participant, showing the video game. The participant performs a stepping movement to play the game

The training is performed using the exergame device Dividat Senso (Dividat AG, Schindellegi, Switzerland, for a detailed description of the device see [85]). The Dividat Senso consists of a TV screen and a pressure-sensitive plate as well as a handrail on three sides to provide security to trainees (Fig. 2). Participants perform stepping movements on the plate to control the games. The system provides real-time feedback on the gamer’s performance, including visual, auditory, and tactile cues facilitating the interaction of the participant with the video games. These video games target different cognitive functions (incl. attentional, executive, memory, and visuospatial functions). Via an online platform, personalised training programmes can be created within the Dividat training system.

### Criteria for discontinuing or modifying allocated interventions {11b}

The intervention is discontinued in case of withdrawal of the informed consent, participant request, or if the health status of the participant or any harm does not allow the continuation of the intervention. Minor individual modifications of the planned intervention are possible (e.g. using a training aid for additional stability) if following the training concept is possible.

### Strategies to improve adherence to interventions {11c}

All sessions are supervised one-to-one by a trained movement scientist, who engages the participant in the study procedures and encourages complying with the set appointments. Additionally, considering each individual’s game preferences is part of the PEMOCS concept’s variability rules to foster motivation and fun during the gaming sessions. Compliance with training sessions and adherence to scheduled training time are recorded.

### Relevant concomitant care permitted or prohibited during the trial {11d}

Concomitant care (usual care) including other therapies is allowed and recorded in both groups during the whole study.

### Provisions for post-trial care {30}

N/A, as there are no disadvantages likely to arise from the intervention.

## Outcomes {12}

**Fig. 3:**
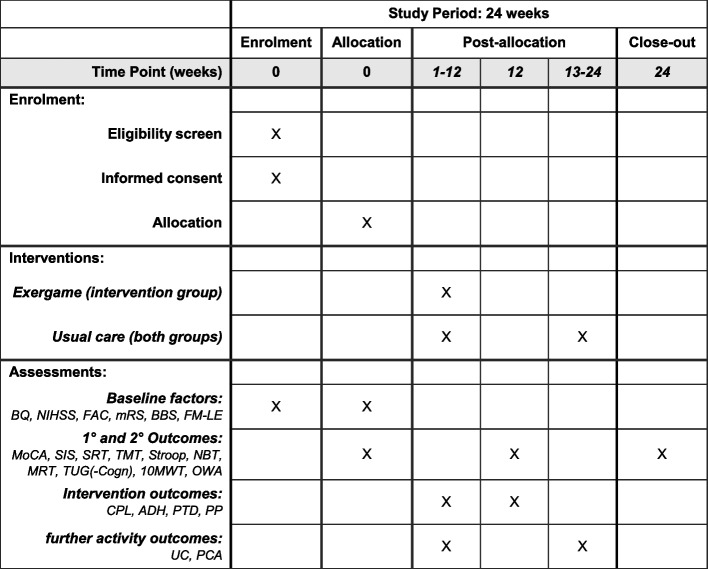
SPIRIT figure. ADH, adherence; BBS, Berg Balance Scale; BQ, baseline questionnaire, incl. see the ‘ Baseline factors’ section; TUG(-Cogn), (cognitive dual-task) Timed Up and Go test; CPL, compliance; FAC, Functional Ambulation Category; FM-LE, lower-extremity component of Fugl-Meyer assessment; MoCA, Montreal Cognitive Assessment; mRS, modified Rankin scale; MRT, mental rotation test; NBT, N-back test; NIHSS, National Institute of Health Stroke Scale; OWA, outdoor walking assessment; PCA, physical and cognitive activities; PP, perceived performance; PTD, perceived task difficulty; SIS 3.0, Stroke Impact Scale; SRT, simple reaction test; Stroop, Stroop Interference test; TMT, Trial Making test; UC, usual care; 10MWT, 10-m walk test

Primary and secondary outcomes are collected at three time points (T0–week 0, T1–week 12, T2–week 24; see Table 2, Figs. 1 and 3) by cognitive and motor assessments as well as a health-related-quality-of-life questionnaire. At the baseline measurement (T0), participant characteristics and baseline factors are recorded. Intervention and activity outcomes are collected during the intervention (T0–T1) and the follow-up (T1–T2) periods, respectively. All variables will be aggregated as means or medians, depending on the distribution of the data, and the analysis metric for all will be final values (see the ‘ Statistical methods’ section). An overview of all outcome variables is presented in Tables 3 and 4.

**Table 2:** Overview over outcome recording. *ADH*, adherence; *BBS*, Berg Balance Scale; *BQ*, baseline questionnaire, incl. see the ‘ Baseline factors’ section; *TUG(-Cogn)*, (cognitive dual-task) Timed Up and Go test; *CPL*, compliance; *FAC*, Functional Ambulation Category; *FM-LE*, lower-extremity component of Fugl-Meyer assessment; *MoCA*, Montreal Cognitive Assessment; *mRS*, modified Rankin scale; *MRT*, mental rotation test; *NBT*, N-back test; *NIHSS*, National Institute of Health Stroke Scale; *OWA*, outdoor walking assessment; *PCA*, physical and cognitive activities; *PP*, perceived performance; *PTD*, perceived task difficulty; *SIS 3.0*, Stroke Impact Scale; *SRT*, simple reaction test; *Stroop*, Stroop Interference test; *TMT*, Trail Making test; *UC*, usual care; *10MWT*, 10-m walk test

Time (weeks)	0	+ 1–12	+ 12	+ 13–24	+ 24
**Time point**	Baseline (T0)	Intervention period	Post-intervention (T1)	Follow-up period	Follow-up (T2)
**Study staff**	Assessor	Training supervisor	Blinded assessor	Training supervisor	Blinded assessor
**Baseline factors:** BQ, NIHSS, FAC, mRS, BBS, FM-LE	+				
**1° Endpoint:** MoCA	+		+		+
**2° Endpoints:** SIS 3.0, SRT, TMT, Stroop, NBT, MRT, TUG(-Cogn), 10MWT, OWA	+		+		+
**Intervention Endpoints:** CPL, ADH, PTD, PP		+ *(intervention group only)*			
**Activity Endpoints:** UC, PCA		+		+

**Table 3:** Overview of primary and secondary outcomes with definitions. *TUG-Cogn* cognitive dual-task Timed Up and Go test, *MoCA* Montreal Cognitive Assessment, *MRT* mental rotation test, *NBT* N-back test, *OWA* outdoor walking assessment, *SIS 3.0* Stroke Impact Scale, *SRT* simple reaction test, *TMT* Trail Making test, *TUG* Timed Up and Go test, *10MWT* 10-m walk test. *The asymmetry index is calculated according to the presented formula not considering affected body side. Allocating left and right in the formula has an impact on the algebraic sign of the asymmetry index, not, however, on its absolute value. As absolute values will be reported for comparability of the asymmetry indices of left- and right-affected patients, the formula can be used as reported [86]. ** [87]

Test	Outcome variables	Definition
**Primary outcome**		
MoCA	Total score (0–30)	= Sum of domain scores + 1 point for participants with ≤ 12 years of education and a MoCA score < 30
**Secondary outcomes**		
SIS 3.0	- Total score (0––100)	= Sum of domain scores
	- Perceived recovery (0–100%)	Collected by visual analogue scale
	- Domain scores (0–100%): strength, memory/thinking, emotion, communication, ADL/IADL, mobility, hand function, participation	$=\frac{\text{Sum of Likert ratings}}{\textrm{Number of items}}\times 100$
SRT	Of all six conditions:	
	- Reaction time	= Logarithmic mean of trial reaction times
	- Missed	Number of non-answered stimuli within 1500 ms
	- Mistakes	Number of reactions upon no stimulus
TMT A&B	- Time A, time B	Processing time of each sub-test
	- Mistakes A, mistakes B	Number of incorrect touches in each sub-test
	- B to A ratio	$=\frac{\text{Time B}}{\textrm{ Time A}}$
Stroop	For reading and naming:	
	- Interference tendency	= Median RT interference – median RT baseline
	Of all four conditions:	
	- Reaction time	= Median reaction time of all trials
	- Mistakes	Number of false reactions
NBT	- Correct	Number of correct responses (stimulus inquired a reaction and participant responded)
	- Omissions	Number of missed responses (stimulus inquired a reaction but participant did not respond)
	- Mistakes	Number of incorrect responses (stimulus inquired no reaction but participant responded)
	- Reaction time (correct)	= Mean reaction time of correct responses
	- Reaction time (mistakes)	= Mean reaction time of incorrect responses
MRT	- Accuracy	$=\frac{\text{Number of correct responses}}{\textrm{ Total number of tasks}}$
	- Reaction time	= Mean reaction time of correct responses
TUG	- Time TUG	= Mean time of the 3 motor single-task trials
	- Type of cognitive task	Serial subtraction or verbal fluency
	- Correct response rate single-task (CRR_single_)	= Mean CRR of the 3 cognitive single-task trials
		CRR $=\frac{\text{Number of correct responses}}{\text{ Time}}$
	- Time TUG-Cogn	= Mean time of the 3 dual-task trials
	- Correct response rate TUG-Cogn (CRR_dual_)	= Mean CRR of the 3 dual-task trials
	- Motor dual-task effect (DTE%_motor_)	$=\frac{\text{Time TUG-Cogn - Time TUG}}{\textrm{Time TUG}}\times 100$
	- Cognitive dual-task effect (DTE%_cognitive_)	$=\frac{{\text{CRRdual-CRRsingle}}}{{\textrm{CRRsingle}}}\times 100$
10MWT,	- Time	= Mean time for 10m of the three trials
preferred	- Gait speed (preferred)	= Mean gait speed
		Instruction: ‘Walk at a comfortable speed’
	- Cadence	= Mean steps/min
	- Stride length	= Mean stride length
	- Stride length variability	= Mean stride length variability
	- Stride time	= Mean stride time
	- Stride time variability	= Mean stride time variability
	- Double support time	= Mean double support time
	- Stance phase affected, unaffected	= Mean stance phase
	- Swing phase affected, unaffected	= Mean swing phase
	- Swing width affected, unaffected	= Mean swing width
	- Asymmetry index*	$=\frac{\text{Swing phaseleft - Swing phaseright}}{\textrm{ 0.5 (Swing phaseleft + Swing phaseright)}} \times 100$
	- Walk ratio**	$=\frac{\text{Mean Corrected Stride Length/2}}{\textrm{ Corrected Cadence}}$
10MWT,	- Time (fast)	= Mean time for 10m of the three trials
fast		Instruction: ‘Walk as fast but safe as possible’
OWA	- Gait speed (preferred)	= Mean gait speed
		Instruction: ‘Walk at a comfortable speed’
	- Cadence	= Mean steps/min
	- Stride length	= Mean stride length
	- Stride length variability	= Mean stride length variability
	- Stride time	= Mean stride time
	- Stride time variability	= Mean stride time variability
	- Double support time	= Mean double support time
	- Stance phase affected, unaffected	= Mean stance phase
	- Swing phase affected, unaffected	= Mean swing phase
	- Swing width affected, unaffected	= Mean swing width
	- Asymmetry index*	s. above
	- Walk ratio**	s. above

**Table 4:** Overview of intervention and further activity outcomes. *CA* cognitive activities, *CT* cognitive therapy, *PA* physical activities, *PT* physical therapy

**Intervention outcomes**	
Compliance rate (overall)	$={\text{Mean}} \frac{\text{Number of attended training sessions}}{\textrm{ Number of offered training sessions}} \times 100$
Compliance rates in each week (1–12)	
Adherence rate (overall)	$={\text{Mean}} \frac{\text{Total attended training time}}{\textrm{ Total offered training time}} \times 100$
Adherence rates in each week (1–12)	
Reasons for not attending or aborting a training session	
Perceived motor-cognitive task difficulty (overall)	Collected by visual analogue scale
Perceived m-c-task difficulty in each week (1–12)	
Perceived performance (overall)	Collected by visual analogue scale
Perceived performance in each week (1–12)	
**Further activities: usual care**	
- Frequency intense physical therapy (PT)	= Mean frequency per week of intense PT
- Volume/week intense PT	= Mean volume per week of intense PT
- Total volume intense PT	= Sum of the volume of all intense PT
- Frequency moderate PT	= Mean frequency per week of moderate PT
- Volume/week moderate PT	= Mean volume per week of moderate PT
- Total volume moderate PT	= Sum of the volume of all moderate PT
- Total volume PT	= Sum of the volume of all PT
- Types of PT	
- Frequency intense cognitive therapy (CT)	= Mean frequency per week of intense CT
- Volume/week intense CT	= Mean volume per week of intense CT
- Total volume intense CT	= Sum of the volume of all intense CT
- Frequency moderate CT	= Mean frequency per week of moderate CT
- Volume/week moderate CT	= Mean volume per week of moderate CT
- Total volume moderate CT	= Sum of the volume of all moderate CT
- Total volume CT	= Sum of the volume of all CT
- Types of CT	
- Total volume other therapies	= Sum of the volume of all other therapies
- Types of other therapies	e.g. massage
**Further activities: general**	
- Frequency intense physical activity (PA)	= Mean frequency per week of intense PA
- Volume/week intense PA	= Mean volume per week of intense PA
- Total volume intense PA	= Sum of the volume of all intense PA
- Frequency moderate PA	= Mean frequency per week of moderate PA
- Volume/week moderate PA	= Mean volume per week of moderate PA
- Total volume moderate PA	= Sum of the volume of all moderate PA
- Total volume PA	= Sum of the volume of all PA
- Frequency intense cognitive activity (CA)	= Mean frequency per week of intense CA
- Volume/week intense CA	= Mean volume per week of intense CA
- Total volume intense CA	= Sum of the volume of all intense CA
- Frequency moderate CA	= Mean frequency per week of moderate CA
- Volume/week moderate CA	= Mean volume per week of moderate CA
- Total volume moderate CA	= Sum of the volume of all moderate CA
- Total volume CA	= Sum of the volume of all CA
- Sedentary time per day	= Mean sedentary time per day

### Baseline factors

At baseline, the following information is recorded to describe the study population (see Table 2):

- Demographics (collected via baseline questionnaire): age, sex, years of education, marital status
- Other characteristics (collected via baseline questionnaire): weight, height, handedness
- Stroke diagnostic details (collected via baseline questionnaire): number of strokes, time point/type (ischemic, haemorrhagic)/lesion site/side and location of initial paresis of the (most recent) stroke, initial (if available), and current (at baseline) National Institutes of Health Stroke Scale (NIHSS [ 88])
- Clinical characteristics A (collected via baseline questionnaire): comorbidities (Charlson Comorbidity Index, CCI [ 89]), modified Rankin scale (mRS[ 90])
- Clinical characteristics B (collected by the assessor at the baseline measurement): Functional Ambulation Category (FAC [ 91]), Lower-Extremity component of the Fugl-Meyer assessment (FM-LE), Berg Balance Scale (BBS [ 92])

### Primary outcome

The primary outcome is global cognitive functioning measured by the total score of the Montreal Cognitive Assessment (MoCA) [93]. The MoCA is composed of several tests assessing different cognitive domains including attention, executive functions, working memory, short-term memory recall, visuospatial skills, and orientation [74]. The MoCA has been successful in detecting cognitive decline [94] and showed good reliability in stroke patients and healthy older adults [95–97] as well as fair to good responsiveness and validity [98] in chronic stroke patients [99]. The maximum achievable score is 30 points, where more points represent better cognitive functioning. For individuals with 12 or less years of education and a total score < 30, an additional point is added [93]. A MoCA score below 24 points indicates mild cognitive impairment in individuals after stroke [74, 100, 101], and an improvement of 1.22 points was found to be a clinically relevant change [99].

Global cognitive functioning, measured by the total score of the MoCA, covers the cognitive domains typically impaired after stroke. Further cognitive tests examining specific cognitive domains/functions will be implemented as secondary outcomes [102].

### Secondary outcomes

Health-related quality of life and perceived recovery are assessed using the total score and the single domain scores of the Stroke Impact Scale [SIS 3.0 [103]]. The SIS 3.0 is a stroke-specific questionnaire assessing the self-reported health status on 5-point Likert scales [98]. It encompasses eight domains (strength, memory/thinking, emotion, communication, ADL/IADL, mobility, hand function, and participation) and a visual analogue scale, where the perceived state of recovery is rated (0 to 100%) [98]. The final score lies between 0 and 100, where a higher score indicates better health-related quality of life. The German SIS (DE-SIS, translated and cross-culturally adapted) was found reliable and valid for the use in German-speaking stroke survivors [104].

Secondary cognitive outcomes include the following computer-based cognitive assessments:

- To assess alertness, a simple reaction test (SRT) (‘WAFA’ within the Vienna Test System, VTS, see the ‘Plans for assessment and collection of outcomes’) is applied. The SRT is a reliable and valid neuropsychological assessment for alertness [105–107]. It composes of six tests with visual and auditory stimuli, of which two evaluate intrinsic alertness (participant has to react to an appearing stimulus as fast as possible), two evaluate crossmodal-phasic alertness (a crossmodal warning stimulus precedes the actual stimulus and the participant has to only react to the actual and not the warning stimulus), and two evaluate unimodal-phasic alertness (a unimodal warning stimulus precedes the actual stimulus and the participant has to only react to the actual and not the warning stimulus) [107].
- Processing speed and cognitive flexibility, a sub-domain of executive functions, are assessed using the Trail Making test (TMT) (‘TMT—Langensteinbacher Version’ within the VTS), which is a widely used, reliable, and valid neuropsychological assessment [108–110]. The TMT consists of two parts: TMT-A assesses general information-processing speed; it asks to connect rising numbers (1–25) as fast as possible. TMT-B is used to test cognitive flexibility; the task is to connect rising numbers and letters alternatingly [111].
- To assess interference inhibition, another sub-domain of executive functions, the Stroop Interference test (‘STROOP’ within the VTS) is used [112]. The Stroop test is a widely used, reliable, and valid neuropsychological assessment testing the ability to inhibit the reaction to a more dominant stimulus in favour of the inquired reaction to a less dominant stimulus [113]. This assessment contains four sub-tests: two baseline and two interference conditions. In the first baseline condition, colour words are presented in grey font and the participant must select the correct colour (baseline reading). In the second baseline condition, coloured bars are presented, and the participant must select the correct colour (baseline naming). In the interference conditions, the colour words are shown in coloured fonts and the participant has to either select the correct colour of the word (interference reading) or of the font (interference naming) [114].
- To assess working memory and related cognitive functions, the N-back test (NBT) (‘NBV’ within the VTS) is used, which is a widely used, reliable, and valid neuropsychological test [115–117]. The participant is presented a row of letters and has to decide upon every letter whether it corresponds to the one shown N letters earlier [118]. In this study, the test condition *N* = 2 is used.
- Mental rotation ability, a sub-domain of visuospatial functions, is assessed using the mental rotation test (MRT) (‘3D’ within the VTS), which is based on the paradigm by Shepard and Metzler [119]. It determines the ability to mentally rotate abstract objects and has been used in stroke patients before [85, 120, 121]. Each item consists of a figure composed of a number of blocks. The participant has to imagine how the arrangement of the blocks looks when viewed from another perspective and choose the correct 2D-view from a selection of four possible solutions [122].

Secondary mobility and dual-task outcomes included the following assessments:

- The Timed Up and Go test (TUG), a reliable and valid assessment in stroke patients [123, 124], is conducted to analyse changes in mobility and dynamic balance. Participants are instructed to perform the TUG ‘as fast and safely as possible’. Time is measured from the moment the participant’s back leaves the backrest of the chair until it touches the backrest of the chair again [125].
- To assess mobility under dual-task conditions and dual-task effects, the TUG-Cognitive is also performed [126]. The TUG-Cognitive (TUG-Cog) is a reliable and valid assessment of dual-task mobility in stroke patients and healthy adults [125, 126]. A cognitive task is first executed in single-task mode during 60 s while being seated on a chair. After this, both tasks, the TUG and the cognitive task, are performed simultaneously, while participants are instructed to not prioritise one over the other. As cognitive task, serial subtraction of 3 from a random number between 50 and 100 (not in the row of three) will be used in participants who are able to complete this task [126, 127]. Participants, who are not able to accomplish serial subtraction, perform a verbal fluency task instead, naming nouns from categories (e.g. fruits, animals, cloths) starting with a specific letter [126, 127]. For all single- and dual-task trials, a familiarisation trial is performed before executing three test trials each. The TUG and TUG-Cog are conducted under laboratory conditions at the participating study centres.

Secondary gait outcomes included a 10-m walk test (10MWT) and an outdoor walking assessment (OWA) using inertial gait sensors to analyse temporal and spatial gait parameters.

- The 10MWT has been found reliable and valid in stroke patients [128]. The 10MWT is performed according to the protocol by Cheng et al. [128], where participants walk 14 m, where only the middle 10 m are timed. The participant starts walking at the 0-m mark, and the stopwatch is started as soon as the first foot crosses the 2-m mark and stopped again when the first foot crosses the 12-m mark, while the participant continues walking to the 14-m mark. At first, a familiarisation trial is performed followed by three trials at comfortable walking and three trials at fast walking speed. Participants are instructed to ‘walk at a comfortable speed’ and ‘walk as fast but safely as possible’, respectively. Participants use their usual walking aid if needed.
- The OWA is a 400-m walk following an outdoor route without stairs [129]. At each study centre, a suitable route nearby was pre-defined and all participants follow this same route. Participants use their usual walking aid if needed and wear proper footwear for an outdoor walk. They are instructed to ‘walk at a comfortable speed, as if they were on a stroll’. To ensure safety, participants are accompanied by two investigators.

### Intervention and further activity outcomes

Compliance and adherence to the trainings and the reasons for not attending or aborting a training session are recorded during the intervention period (T0–T1, see Table 2, Figs. 1 and 3). Additionally, participants in the intervention group are asked to rate the motor-cognitive task difficulty of the training tasks (perceived task difficulty, PTD) and their motor-cognitive performance (perceived performance, PP) in every training session (during T0–T1, see Table 2, Figs. 1 and 3). Visual analogue scales (VAS) in the eCRF based on the cognitive load theory [130] and the NASA-TLX [National Aeronautics and Space Administration Task Load Index [131]] are used to collect these ratings (Additional file 1). PTD and PP are expressed as percentage values where higher percentages stand for more difficult tasks and better performance, respectively. The intervention outcomes are summarised in Table 4.

All participants are interviewed weekly throughout the whole study (T0–T2, see Table 2, Figs. 1 and 3) regarding the dose (frequency and time) and content (intensity and type) of moderate to intense physical and cognitive activities, which they perform as part of their usual care or in their leisure time. Definitions for moderate to intense activities are based on the World Health Organisation’s (WHO) 2020 Guidelines on Physical Activity and Sedentary Behaviour [132]. The interviews are done using a structured questionnaire (Additional file 1), which implies the FITT-VP principles [70] and the TIDieR checklist [Template for Intervention Description and Replication [133]]. The further activity outcomes are summarised in Table 4.

## Participant timeline {13}

Each participant is involved for approximately 24 weeks (12 weeks intervention period, 12 weeks follow-up period, Figs. 1 and 3). Participants are contacted and screened for eligibility by their therapist, physician, and/or the study team. Eligible, potential participants are provided with detailed study information in oral and written form. Interested potential participants are invited to a first study appointment, where they are first again provided with the study information, especially outlining the benefits, risks, and their rights associated with the study, and can clarify remaining questions. Trained movement scientists and therapists then obtain written informed consent from those willing and able to participate in the study, before any study-related procedures start. After that, participants first attend the baseline measurement (T0, Figs. 1 and 3). Subsequently, the participant is allocated randomly to one of the two study arms. Randomisation for each participant is run in Research Electronic Data Capture (REDCap) by an investigator other than the blinded assessor. Participants allocated to the intervention group thereafter attend concept-guided, personalised motor-cognitive training, twice a week for 12 weeks (T0–T1). Participants allocated to the control group receive no additional intervention during the same 12 weeks (T0–T1). After completion of the intervention period, all participants attend the post-intervention measurement (T1, Figs. 1 and 3). During the subsequent 12-week follow-up period (T1-T2), participants in both groups receive no additional intervention. At the end of this period, all participants attend the follow-up measurement (T2).

## Sample size {14}

A sample size of 38 participants (approx. 19 per group) was estimated for this study. The sample size estimation was based on systematic reviews investigating the effects of motor-cognitive training and exergames on cognitive functions. Stanmore et al. included studies with any population (including five out of seventeen studies with stroke or other neurological patients) and found a small to medium effect for global cognitive functioning (SMD = 0.44, *p* = 0.001) and several small to large effects for cognitive domains including executive functions, processing speed and visuospatial skills (0.26 ≤ SMD ≤ 0.90, *p* < 0.05) (132). Five further reviews included studies with older adults and found small to large effect sizes for global or overall cognitive functioning and cognitive domains including attention, executive functions, learning and memory, and processing speed (0.30 ≤ SMD ≤ 1.37, *p* < 0.05) [31, 35, 54, 56, 134]. Based on this evidence, a small to medium effect on global cognitive functioning is anticipated for the planned study (*f* = 0.21). The sample size was estimated using G*Power, entering this effect size (requiring no expected difference between groups and standard deviations) and the following parameters into the mask for a two-way mixed ANOVA; α-level = 0.05, power = 0.80, number of groups = 2, number of measurements = 3, correlation among rep measures = 0.5, nonsphericity correction = 1. Dropouts will be replaced by post-recruitment until the planned sample size is achieved. Based on the recent feasibility study [85] and comparable literature [135–137], a dropout rate of 10–20% can be expected.

## Recruitment {15}

The PEMOCS study is a single-blind randomised control trial (RCT) with chronic stroke survivors recruited from hospitals and rehabilitation centres in the Canton of Zurich, Switzerland. Participants are recruited by therapists and physicians during therapy sessions and stroke follow-up appointments, by flyers on the ward, and by contact of the study team in case of provided general consent. Regular contact between the recruiters and study team meetings should help to maintain an acceptable recruitment rate.

## Assignment of interventions: allocation

### Sequence generation {16a}, concealment mechanism {16b}, and implementation {16c}

The randomisation is stratified by cognitive status (cognitive impairment absent or present, determined by a MoCA score ≥ 24 or < 24, respectively [74, 100, 101]) and by sex (female or male [138–140]). Both stratifying variables are allocated 1:1 to both groups. Participants are randomised using REDCap, the same tool as utilised for eCRF keeping (see the ‘Data management’ section [141, 142]). To perform the randomisation in REDCap, a pre-defined randomisation list in the form of an excel document needs to be uploaded onto the platform, which is then being used by the software. Instructions by REDCap show how this list must be structured to provide the desired allocation ratio and stratification [141, 142]. These instructions were followed by the randomisation-list creator, a person otherwise not involved in the study. This way it was ensured that no member of the study team would know the allocation sequence. After creating the list, the randomisation-list creator encrypted the excel document containing the list with a password. Both, the list and the password, are stored in a secure place, where the investigators of the study team have no access. Moreover, the REDCap user rights to access and view the randomisation setup feature were removed from all investigators of the study team before the final randomisation list was uploaded onto the REDCap platform [141, 142].

Study investigators other than the blinded assessor enrol participants and allocate them to groups. A separate instrument in REDCap, which is invisible for the assessor through user privileges, is used for randomisation.

## Assignment of interventions: blinding

### Who will be blinded {17a}

Outcome assessors are blinded to group allocation. They cannot access the randomisation tool in REDCap and are not involved in intervention procedures. To blind the data analyst, a de-identified dataset will be used for analysis, which will not contain unique identifiers such as the study ID.

### Procedure for unblinding if needed {17b}

N/A, as assessors, who are the only blinded members of the study team, are always accompanied by a training supervisor, who knows the participants and their group allocation. Hence, in case any situation during a measurement session would require knowledge of group allocation, the training supervisor can handle it and the assessor does not need to be unblinded.

## Data collection and management

### Plans for assessment and collection of outcomes {18a}

All outcome variables are gathered in an eCRF (see the ‘Data management’ section). All study-team members gathering data receive specific training for the relevant study procedures. Primary and secondary outcomes are collected at baseline (T0), post-intervention (T1), and follow-up (T2) measurements. The MoCA (primary outcome) is executed on paper following the instructions of the providers (mocacognition.com [93]), and the data is transferred into the eCRF. All assessors obtain a MoCA certificate (mocacognition.com) for trained execution of the test before performing the assessment in the study. The SIS 3.0 is collected via an online questionnaire filled out by the participants electronically in the eCRF or, in case not able to do so, on paper and transferred to the eCRF by the investigators. Secondary computer-based cognitive assessments are conducted within the Vienna Test System (VTS, Schuhfried GmbH, Mödling, Austria), a valid and reliable software for neuropsychological testing. All assessments include written instructions and practice sets and are only started if the participant received clarifying responses on any questions regarding the test functionality. The tests are performed on a touch-screen computer, using either one button on the keyboard or the touch screen to answer the stimuli. All cognitive outcome variables are obtained from the VTS result sheets and transferred into the eCRF. Outcome variables for the TUG and TUG-Cog are collected using a stopwatch and by noting correct answers on paper and directly entered into the eCRF. For the TUG, TUG-Cog, and the 10MWT, practice trials are performed before the actual assessment to ensure clarity of the procedure. Outcome variables of the gait assessments are gathered using the Gait Up system (Gait Up SA, Lausanne, Switzerland) with Physilog® sensors (wearable standalone movement inertial sensors, 50 × 37 × 9.2 mm, 19 g). The Gait Up system provides quantitative, objective, and valid assessment of gait movement [143] presented on output sheets, from where the data are transferred into the eCRF. The first and last two gait cycles are excluded from the analysis to eliminate acceleration and deceleration [86]. Baseline characteristics (see the ‘ Baseline factors’ section) are collected via a questionnaire filled out by the participants electronically in the eCRF or, in case not able to do so, on paper and transferred to the eCRF by the investigators. The results of FMA-LE, FAC, and BBS (see the ‘ Baseline factors’ section) are directly entered into the eCRF. ‘ Intervention and further activity outcomes’ are collected within the eCRF throughout the intervention (T0–T1) and follow-up (T1–T2) periods, respectively. In all phases, deviations from the protocol are recorded in the eCRF to ensure traceability and the ability to exactly repeat the assessment procedures at T0, T1, and T2 for each individual participant.

### Plans to promote participant retention and complete follow-up {18b}

During the follow-up period, participants in both groups are contacted once a week to inquire their usual care, general physical, and cognitive activities (see ‘Intervention and activity outcomes’). This keeps them engaged in the study procedures and, therefore, promotes successful retention.

### Data management {19}

An electronic case report form (eCRF) is kept for each enrolled participant using REDCap electronic data capture tools hosted at ETH Zurich [141, 142]. REDCap (Research Electronic Data Capture) is a secure, web-based software platform designed to support data capture for research studies, providing (1) an intuitive interface for validated data capture, (2) audit trails for tracking data manipulation and export procedures, (3) automated export procedures for seamless data downloads to common statistical packages, and (4) procedures for data integration and interoperability with external sources [141, 142]. This eCRF has been validated before enrolment of the first participant. Study team members who are authorised to enter or edit data in the eCRFs, receive a login to the REDCap study platform, and are listed with signatures in the trial master file (TMF) and the investigator site file (ISF). To assure that any authorised person, who may perform data entries and changes in the eCRF, can be identified, all entries/edits are recorded with name, date, and time. Data entry of the primary and secondary outcomes in REDCap is performed by one and double-checked by another investigator (verification, four-eyes-principle). Should any previously entered data need to be changed (e.g. because a mistake was identified during the verification), a reason must be given to proceed. eCRFs are kept current to reflect participant status at each phase during the study.

Study and participant data will be handled with uttermost discretion and are only accessible to authorised personnel who require the data to fulfil their duties within the scope of the study. Participants are coded and not identifiable in the eCRF or on any other study-specific documents. Appropriate coded identification (study ID) is used. Each study ID composes of four random letters or numbers, which are not related to any characteristics, or the time point of inclusion of the participants. The sponsor will store the participant identification list in a secured and locked location. All study data are archived for ten years after study termination or premature termination of the study.

### Confidentiality {27}

Personal information of potential and enrolled participants is kept confidential and only accessible for involved study-team members for study-related purpose. Data protection is kept according to current guidelines of the Swiss law. Participants, who have withdrawn from the study, can ask the deletion of their personal information at any time. All participants receive a study ID (a random sequence of four letters and numbers) not associable with their personal data. All study data is stored only with this ID and never related to any personal data. The key to decode study data is kept locked and only accessible for involved study-team members for study-related purpose(s). After completion of the study procedures, study data are archived according to Good Clinical Practice (GCP) guidelines for at least 10 years.

### Plans for collection, laboratory evaluation, and storage of biological specimens for genetic or molecular analysis in the trial/future use {33}

N/A as no biological specimens are collected in this study.

## Statistical methods

Microsoft Excel (Microsoft Corporation, 2016) will be used to aggregate and tabulate the data. All statistical analyses will be performed using RStudio open-source software (Bosten, USA [144]) or SPSS Statistics (version 26 for windows; IBM, Chicago, IL, USA).

### Statistical methods for primary and secondary outcomes {20a}

Distributions of all baseline factors and primary and secondary outcome variables will be checked with the Shapiro–Wilk test [145]. Appropriate descriptive statistics will be obtained for all baseline factors and outcome variables (means and standard deviations for normally distributed data, medians and inter-quartile ranges for non-normally distributed data, frequencies for categorical data). Differences between groups in baseline factors will be evaluated using an independent *t*-test if assumptions for parametric testing are met or a non-parametric alternative otherwise. For categorical data, a chi-square test or a Fisher’s exact test will be used as appropriate [146].

Assumptions on the residuals of primary and secondary outcomes will be checked using the DHARMa package in R [147]. Appropriate actions will be taken if one or more assumptions are not met. All primary and secondary outcomes will be analysed following the standard intention-to-treat (ITT [148, 149]) principle using linear mixed-effects models (LMEM, lme4 package in R). Subject-specific random intercepts will account for within-subject correlations between time points. Follow-up scores (T2) of the outcomes will be the dependent variables of the models, while group (intervention vs. control, control being the reference), time (T0, T1, T2), and group x time interactions at T1 and at T2, respectively, will be included as independent variables (fixed effects). Baseline factors such as age, sex, and time since stroke will be considered as covariates. Missing data of whole measurement time points (i.e. of dropouts) will be accounted for with the ‘last observation carried forward’ method. For single missing data points (e.g. if a participant did not perform an assessment at one time point or a technical issue produced missing data at one time point), however, no data imputation will be performed as LMEMs can be fitted even if some outcome data are missing [145, 150]. For the outdoor walking assessment (OWA), high occurrence of missing data is expected, as ability to walk 400 m is not covered by the eligibility criteria and varying weather conditions on the measurement days may interfere with the assessment. Therefore, for the OWA outcome parameters, datasets of participants who did not perform the OWA at one or several time points will be excluded from the analysis. Significance will be set to *p* < 0.05. Effect sizes will be calculated as *r* (Bravais-Person correlation coefficient) and interpreted as small (*r* < 0.3), medium (*r* < 0.5), and large (*r* ≥ 0.5) [145].

### Methods for analysis of intervention and further activity outcomes

Mean/median compliance and adherence rates with standard deviations/inter-quartile ranges will be reported overall and for each week of the intervention period. Additionally, reasons for not attending or aborting a training session will be summarised. Mean/median ratings of perceived motor-cognitive task difficulty and perceived performance with standard deviations/inter-quartile ranges will be reported overall and for each week of the intervention period. These will be compared to the targeted ranges for task difficulty and perceived performance to establish if an optimal training load was achieved. The volume of usual care (physical, cognitive, and other therapies) and general physical as well as cognitive activities will be descriptively summarised and considered as covariates in the LMEMs of the primary and secondary analyses.

### Interim analysis {21b}

N/A. No interim analyses are planned, as preliminary analysis of the effect will most probably be underpowered and, therefore, not informative for the decision of an early study termination.

### Methods for additional analysis (e.g. sub-group analyses) {20b}

As the ITT analyses may underestimate a present treatment effect [148], the analyses of the primary and secondary outcomes will be repeated with only those participants, who did not withdraw from the study within the intervention period (T0–T1), and with adherence rates of ≥ 85% (per protocol analysis). This cut-off was chosen based on systematic reviews covering comparable interventions, outcomes, and populations [27, 151], where a minimum of 720 min in at least 12 weeks or an intervention duration of at least 8 weeks were recommended. Moreover, 85% of 24 training sessions results in an ‘acceptable’ absence of 3.6 sessions, which seems practical to account for sickness and conflicting schedules. The results of the ITT and per-protocol analyses will be compared in the discussion of the study report.

Furthermore, to assess clinical meaningfulness of possible treatment effects, the following analyses will be performed on outcomes that (a) revealed a significant between group effect at either T1 or T2 [152, 153] and (b) a clinically important difference is reported in literature. On the one hand, the difference in change score between the two groups will be compared to clinical meaningful change scores (e.g. 1.22 points in the MoCA [99]). On the other hand, frequencies of ‘responders’ (individual change score above clinically meaningful change) and ‘non-responders’ (individual change score below clinically meaningful change) between the two groups will be compared [152, 153].

### Plans to give access to the full protocol, participant-level data, and statistical code {31c}

The full protocol of this study was published on https://clinicaltrials.gov (NCT05524727). De-identified participant-level data and the statistical code will be available from the corresponding author on reasonable request.

## Oversight and monitoring

### Composition of the coordinating centre and trial steering committee {5d}

Recruitment and screening are performed at all study cites by the (local) principal investigators or their delegated staff. All other study-related procedures are performed by trained members of the sponsor/principal investigator team, who are in daily contact regarding the organisation of the trial.

### Composition of the data monitoring committee, its role and reporting structure {21a}

Data monitoring in this study is performed by a senior researcher not otherwise involved in the study procedures. On at least three monitoring visits, the monitor reviews the study (team) documents and regulatory aspects, the enrolment process, the participant data, safety aspects, and protocol deviations. Upon monitoring visits, the monitor generates a report including any findings that must be resolved. Resolution of these findings is performed by the principal investigator. Additionally, the clinical trial centre (CTC) of the University Hospital Zurich performs a quality visit on the protocol and monitoring reports. Both the monitor and the clinical trial centre are independent of the sponsor.

### Adverse event reporting and harms {22}

Adverse events are recorded in the eCRF throughout the study and managed according to GCP guidelines.

### Frequency and plans for auditing trial conduct {23}

The project management group (including study staff from all sites) meets at least weekly to track correct trial conducted. As this is a low-risk study, no data monitoring committee exists; however, according to the approved study protocol by the cantonal ethical committee, an independent monitor performs at monitoring visits on the study documentation and procedures, including but not limited to the following actions:

1. An initiation visit before the study start, where is evaluated whether ethical approval is granted, training of study staff was performed and documented, study and safety documentation as well as case report forms were appropriately prepared, and insurance is valid;
2. At least one routine monitoring visit (further visits are planned if indicated due to inconveniences), where it is checked whether protocol and study documentation is up-to-date, informed consent procedures and enrolment are correctly performed, source data is correctly filed and data transferred to case report forms, safety is correctly reported and treated, and blinding has been maintained; and
3. A closure visit upon the end of the study, where the monitor repeats the checks from the routine monitoring visit, and additionally assesses whether documentation is complete and archiving appropriately prepared.

### Plans for communicating important protocol amendments to relevant parties (e.g. trial participants, ethical committee) {25}

Necessary protocol amendments are submitted to the ethical committee before implementation. All study-team members are informed about changes to the protocol on the weekly project management group meetings. Updated versions of the protocol are added to the investigator site files upon approval of the ethical committee. In case of an amendment, which changes study procedures or conditions for participants, participants are informed immediately and the clinical trial registry is updated upon approval of the ethical committee.

## Dissemination plans {31a}

The findings of this study will be published in scientific journal articles and scientific presentations. All publications will be authored by the study team, following established authorship guidelines. Participants will receive a copy of their individual study data upon request.

## Discussion

The PEMOCS study evaluates the effect of a 12-week concept-guided, personalised, motor-cognitive exergame training added to usual care compared to usual care alone on global cognitive functions and explores effects on specific cognitive functions, health-related quality of life, and gait in chronic stroke survivors. The results will give insight into under-investigated research topics in chronic stroke: the effect of a motor-cognitive exergame integrating whole-body movements on cognitive functions, its effect on spatiotemporal gait parameters relevant in stroke, and the benefits of personally-tailored progression and variability in exergame interventions [37]. The feasibility trial preceding this RCT showed that chronic stroke survivors were continuously motivated for and satisfied with the personalised exergame training and adherence was high [85]. Therefore, satisfactory adherence in the training group is expected in this study. Based on results from studies with exergame trainings in healthy older adults or other neurological patients, superior effects on cognitive functions of the additional exergame training compared to usual care alone are hypothesised [34, 63, 154]. We expect most participants with chronic stroke to exhibit cognitive decline, as cognitive impairment is widely reported in chronic stroke, even in patients with seemingly good clinical outcome [155, 156]. We found significantly lower MoCA values in a high-functioning stroke sample compared to healthy adults with comparable age in a previous study [129]. We did not, however, include a MoCA-based threshold for cognitive impairment for inclusion because such a screening instrument seems unsuitable to identify subtle or specific cognitive deficits [76]. Furthermore, previous research has shown that exergame training may have positive effects on gait and mobility [37, 67], including gait speed, spatiotemporal gait parameters, and mobility in chronic stroke. Therefore, superior improvements in various spatiotemporal gait parameters and mobility in the intervention compared to the control group are expected in this study.

The findings of this study could be considered for the design and prescription of future long-term rehabilitation interventions for chronic stroke survivors that focus on cognitive functioning next to restoring motor functions. This is an identified need since cognitive dysfunction after stroke has a persistently high prevalence [157–159]. Exergames have the potential to increase motivation for training [42, 85], to produce additional benefits on physical functions as compared with conventional care modes [60, 67], as well as on cognitive functions [55, 63, 154]. Exergames that contain aspects of virtual reality have shown to be able to significantly effect on *Body Structure/Function* and *Activity* level outcomes, including improvements in cognitive function, and there is, therefore, evidence supporting the use of such interventions as an adjunct for stroke rehabilitation [160, 161]. Therefore, exergames could help expand existing rehabilitation services for chronic stroke survivors, which may lead to further improvements of cognitive and motor functioning and, therefore, contribute to increased quality of life after stroke [162].

Due to its design, this study will have two major limitations. (1) Study participants will train 840 min with 100% adherence rate, which is at the lower border of recommended training volume for improving cognitive functions in chronic stroke and older adults [23, 27, 163, 164]. Similarly, some authors recommend more than two and longer sessions per week [55, 164]. However, practical reasons including that participants needed to come into the study centre twice a week limited more training time in this study. Moreover, the feasibility study preceding this RCT showed that participants preferred two sessions per week, lasting 30 to 40 min and no longer [85]. To prevent over-request of time expenditure of the participants and, thereby, harm recruitment, the total training volume was kept at this lower border of recommended time. (2) The black box of usual care and leisure time physical and cognitive activities may influence the measured outcomes as well. Usual care is a wide term and has been reported to include a wide range of interventions, doses, intensities, and implementations [165, 166]. This limitation is addressed by recording the participants’ usual care and general activities besides the study intervention and by considering this in the analysis. (3) This study’s sample size was not based on a standard RCT sample size calculation, as at the time of planning, no expected values for difference between groups and standard deviations for the MoCA in chronic stroke were available due to the novelty of the topic in research. Therefore, the sample size was estimated based on effect sizes from systematic reviews in related literature (see the ‘Sample size’ section).

## Trial status

The protocol version 2 of this study was approved by the local Ethics Committee in Switzerland (Ethics Committee of the Canton Zurich, project-ID: 2022–01211) in August 2022. Since then, three amendments were submitted to the ethics committee: (1) protocol version 3 (October 2022) enclosed a change in the patient information regarding travelling costs to study centres, (2) protocol version 4 (March 2023) reported a new principal investigator at one of the study sites, and (3) protocol version 5 (October 2023) included a more detailed description of the data management, which was suggested by the monitor. None of the amendments changed any of the study procedures. The current version 5 of the protocol was approved by the Ethics Committee on October 20, 2023. Study procedures began in September 2022 and are expected to be completed by July 2024. At the time point of submission of this manuscript, the study is running. At submission of this manuscript, 37 participants were enrolled and planned to completed the study or had already completed it. Nine additional participants had been enrolled but withdrew before study completion. The study was registered on https://clinicaltrials.gov (NCT05524727) on September 1, 2022, as well as on https://.kofam.ch, the portal for human research in Switzerland, which ensures that none of the study procedures have been changed since the start of the study.

### Supplementary Information

Supplementary Material 1.

## Data Availability

No preliminary clinical results will be published before the end of the trial. The final trial dataset with anonymous data will be available on a repository or included with the publication as additional file.

## Abbreviations

ADH: Adherence
ANOVA: Analysis of variance
BBS: Berg Balance Scale
BQ: Baseline questionnaire, incl. see the ‘Baseline factors’ section
CA: Cognitive activity
CCI: Charlson comorbidity index
CPL: Compliance
CT: Cognitive therapy
eCRF: Electronic case report form
FAC: Functional Ambulation Category
FITT-VP: Frequency, intensity, time, type, volume, progression
FM-LE: Lower-extremity component of the Fugl-Meyer assessment
GCP: Good clinical practice
ISF: Investigator side file
LMEM: Linear mixed-effects model
MoCA: Montreal Cognitive Assessment
mRS: Modified Rankin scale
MRT: Mental rotation test
NASA-TLX: National Aeronautics and Space Administration Task Load Index
NBT: N-back test
NIHSS: National institutes of health stroke scale
OWA: Outdoor walking assessment
PA: Physical activity
PCA: Physical and cognitive activities
PI: Principal investigator
PP: Perceived performance
PT: Physical therapy
PTD: Perceived task difficulty
RCT: Randomised, controlled trial
REDCap: Research Electronic data capture
(S)AE: (Serious) adverse event
SIS 3.0: Stroke Impact Scale
SMD: Standardised mean difference
SRT: Simple reaction test
Stroop: Stroop Interference test
T0: Baseline
T0-T1: Intervention period
T1: Post-intervention
T1-T2: Follow-up period
T2: Follow-up
TIDieR: Template for intervention description and replication
TMF: Trial master file
TMT: Trail Making test
TUG: Timed Up and Go test
TUG-Cogn: Cognitive dual-task Timed Up and Go test
UC: Usual care
VAS: Visual analogue scale
VTS: Vienna Test System
10MWT: 10-M walk test
