# Supplementary material for: PEMOCS: Evaluating the effects of a concept-guided, PErsonalised, MOtor-Cognitive exergame training on cognitive functions and gait in chronic Stroke—study protocol for a randomised controlled trial
Source: Trials. 2024 Jul 4;25:451. doi: 10.1186/s13063-024-08283-7 (PMC11223407; doi:10.1186/s13063-024-08283-7)
Supplement: Supplementary file 1 — Supplementary Material 1. [file 13063_2024_8283_MOESM1_ESM.pdf]

Additional file 1

1. Visual analogue scales used to collect ratings of Perceived Performance (A) and Perceived Task Difficulty (B).

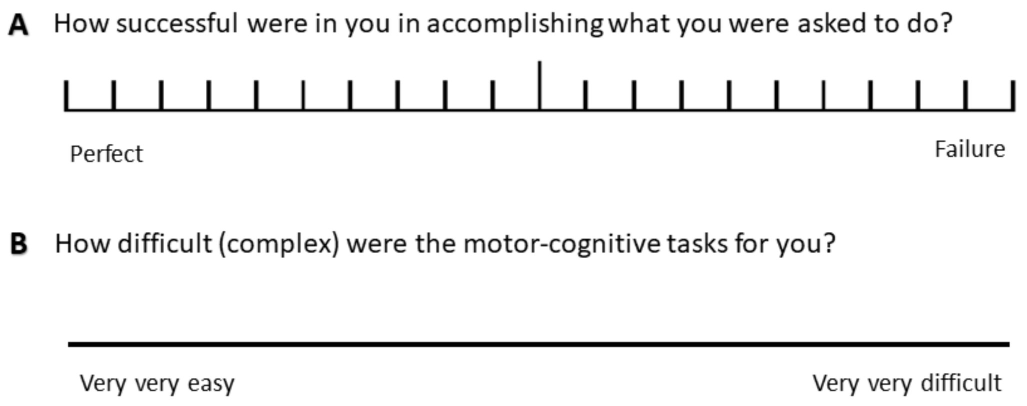

A: The scale for perceived performance is based on the performance sub-score of the NASA Taskload Index [NASA-TLX, (Flagel et al. 2019)].

B: The scale for perceived task difficulty is based on the scale for cognitive load presented in the Cognitive Load Theory (Sweller 2011).

2. Questionnaire for Physical and Cognitive Activities and Usual Care

Part. ID: **Date:** **Week:**

1. Questions on Physical and Cognitive Activities

Physical Activity:

In the previous week, did you perform intensive physical activity or exercise (e.g. jogging), where breath and heart rate increased strongly, of at least 10 minutes?

☐ No ☐ Yes, on ___ (number) week days in total approx. __:__ (h:min)

In the previous week, did you perform moderate physical activity or exercise (e.g. brisk walking, strength training), where breath and heart rate increased slightly, of at least 10 minutes?

☐ No ☐ Yes, on ___ (number) week days in total approx. __:__ (h:min)

How much time did you spend sitting or resting (e.g. at a desk, sitting with friends, riding a car / train, watching TV)?

On average ___:___ (h:min) per day

Cognitive Activity:

In the previous week, did you perform intensive cognitive activity (e.g. memory or reaction training), of at least 10 minutes?

☐ No ☐ Yes, on ___ (number) week days in total approx. __:__ (h:min)

In the previous week, did you perform moderate physical activity (e.g. puzzle solution, strategy games), of at least 10 minutes?

☐ No ☐ Yes, on ___ (number) week days in total approx. __:__ (h:min)

5. Questions on Usual Care

Physical Therapy:

In the previous week, did you receive intensive physical or exercise therapy, where breath and heart rate increased strongly (e.g. physical, occupational or training therapy)?

☐ No ☐ Yes, on ___ (number) week days in total approx. __:__ (h:min)

If yes, what type? ____

If yes, who provided it? ☐ physical / occupational / exercise therapist
☐ other person: _______________________

In the previous week, did you receive moderate physical or exercise therapy, where breath and heart rate increased slightly (e.g. physical, occupational or training therapy)?

☐ No ☐ Yes, on ___ (number) week days in total approx. __:__ (h:min)

If yes, what type? ____

If yes, who provided it? ☐ physical / occupational / exercise therapist
☐ other person: _______________________

In the previous week, did you received another type of physial therapy (e.g. massage, lymph drainage)?

☐ No ☐ Yes, on ___ (number) week days in total approx. __:__ (h:min)

If yes, what type? ______________________________

If yes, who provided it? ______________________________

Cognitive Therapy:

In the previous week, did you receive cognitive therapy (e.g. occupational therapy, neuropsychology)?

☐ No ☐ Yes, on ___ (number) week days in total approx. __:__ (h:min)

If yes, what type? ______________________________

If yes, who provided it? ☐ occupational therapist / neuropsychologist
☐ other person: ______________________

Did you receive any other type of therapy in the previous week?

☐ No ☐ Yes, on ___ (number) week days in total approx. __:__ (h:min)

If yes, what type? ______________________________

If yes, who provided it? ______________________________

Questions were based on the definitions for moderate and intense activities by the World Health Organisation [WHO, (Bull et al. 2020)].

References

Bull, F. C., S. S. Al-Ansari, S. Biddle, K. Borodulin, M. P. Buman, G. Cardon, . . . J. F. Willumsen (2020). "World Health Organization 2020 guidelines on physical activity and sedentary behaviour." Br J Sports Med **54**(24): 1451-1462 DOI: 10.1136/bjsports-2020-102955.

Flagel, K., B. Galler, J. Steinhauser and K. Gotz (2019). "[The "National Aeronautics and Space Administration-Task Load Index" (NASA-TLX) - an instrument for measuring consultation workload within general practice: evaluation of psychometric properties]." Z Evid Fortbild Qual Gesundhwes **147-148**: 90-96 DOI: 10.1016/j.zefq.2019.10.003.

Sweller, J. (2011). Cognitive load theory. Psychology of learning and motivation, Elsevier. **55:** 37-76.
